# The role of telomeres and telomerase in hematologic malignancies and hematopoietic stem cell transplantation

**DOI:** 10.1186/s13045-014-0061-9

**Published:** 2014-08-20

**Authors:** Limengmeng Wang, Haowen Xiao, Xing Zhang, Chong Wang, He Huang

**Affiliations:** 1 Bone Marrow Transplantation Center, The First Affiliated Hospital, Zhejiang University, School of Medicine, No. 79 Qingchun Rd, Hangzhou 310003, Zhejiang Province, P R China; 2 Department of Haematology, Guangzhou Liuhuaqiao Hospital, Guangzhou, Guangdong Province, P R China; 3 Department of Haematology, The First Affiliated Hospital, Zhengzhou University, Zhengzhou, Henan Province, P R China

**Keywords:** Telomere, Telomerase, Shelterin, Hematologic malignancies, Hematopoietic stem cell transplantation, Target therapy

## Abstract

Telomeres are specific nucleoprotein structures at the ends of eukaryotic chromosomes. Telomeres and telomere-associated proteins maintain genome stability by protecting the ends of chromosomes from fusion and degradation. In normal somatic cells, the length of the telomeres gradually becomes shortened with cell division. In tumor cells, the shortening of telomeres length is accelerated under the increased proliferation pressure. However, it will be maintained at an extremely short length as the result of activation of telomerase. Significantly shortened telomeres, activation of telomerase, and altered expression of telomere-associated proteins are common features of various hematologic malignancies and are related with progression or chemotherapy resistance in these diseases. In patients who have received hematopoietic stem cell transplantation (HSCT), the telomere length and the telomerase activity of the engrafted donor cells have a significant influence on HSCT outcomes. Transplantation-related factors should be taken into consideration because of their impacts on telomere homeostasis. As activation of telomerase is widespread in tumor cells, it has been employed as a target point in the treatment of neoplastic hematologic disorders. In this review, the characteristics and roles of telomeres and telomerase both in hematologic malignancies and in HSCT will be summarized. The current status of telomerase-targeted therapies utilized in the treatment of hematologic malignancies will also be reviewed.

## Introduction

The telomeres are specific nucleoprotein structures at the ends of eukaryotic chromosomes which maintain genome stability by protecting chromosomes from end fusion and degradation. Human telomeres are composed of 10–15 kb of 5′-TTAGGG-3′ DNA sequence repeats and a telomere-associated protein complex, shelterin (reviewed by Blackburn) [1]. The end of each telomere consists of a t-loop structure formed by strand invasion of the 3′ single strand overhang into the double-stranded telomeric DNA and then stabilized by shelterin [2].

In most somatic cells, telomeres gradually become shortened (20–59 bp/year) because of the end-replication problem during cell division [3],[4]. Once its length reaches a critical limitation, the telomere is unable to assemble the t-loop structure and the chromosome becomes uncapped. At this point the DNA damage response and replicative senescence will be triggered through the ataxia telangiectasia-mutated gene (ATM) or the ataxia telangiectasia and Rad3 (ATR) -related checkpoint pathway [5]. However, cells in which cell cycle checkpoint proteins have been inactivated are able to continue division and continue losing telomeric sequences until they reach a crisis stage in which p53-dependent apoptosis is triggered. Cancer cells have to go through the crisis stage to maintain their telomeres and achieve immortality. In the majority of cancer cells (80% to 90%), telomerase has been activated to maintain telomere length [6], while a subset of cancer cells elongate telomeres through telomerase-independent mechanisms named alternative lengthening of telomeres (ALT) [7].

Telomerase is a reverse transcriptase which maintains telomere length by adding nucleotides to the single-stranded (ss) DNA of the telomere during cell division [8]. Telomerase consists of a protein component (hTERT) and an RNA template component (hTERC). hTERT is the catalytic subunit of telomerase which limits its reverse transcriptase activity. hTERC consists of an 11 nucleotide sequence (5′-CUAACCCUAAC-3′) which is complementary to the telomere sequence (TTAGGG)n [9]. Telomerase is recruited to the telomere via its interaction with shelterin.

**Figure 1:**
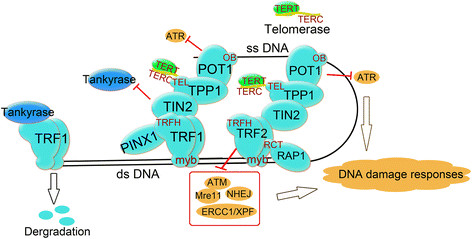
**The structure of shelterin.** TRF1 and TRF2 bind to double strand (ds) DNA and form two separate complexes with other shelterin proteins. POT1 binds to single strand (ss) DNA while RAP1 binds to DNA at the ds-ss joint. PINX1 is a TRF1 binding protein. TIN2 binds TRF1 and TRF2 spontaneously and protects TRF1 from being degraded by tankyrase. TPP1 and POT1 form a heterodimer. TPP1 links TIN2 and POT1/TPP1 and recruits telomerase to the shelterin complex.

Shelterin is a protein complex which consists of 6 telomere-associated proteins: telomeric repeat-binding factors 1 and 2 (TRF1 and TRF2), TRF1-interacting nuclear factor 2 (TIN2), protection of telomeres (POT1), POT1 and TIN2-interacting protein 1 (TPP1), and TRF2-interacting protein 1 (Rap1) (Figure 1). Shelterin protects the telomere from being recognized as a double-strand break in the DNA sequence which would lead to activation of the DNA damage response and repair process (reviewed by de Lange) [10]. Shelterin is anchored to the chromosomal end by the double-strand (ds) DNA binding proteins TRF1 and TRF2, the ss DNA binding protein POT1, and Rap1 which binds DNA at the ds-ss junction of the telomere [11]. TRF1 and TRF2 bind TIN2 simultaneously to form two separate complexes [12],[13]. The binding between TRF1/TRF2 and TIN2 protects TRF1/TRF2 from degradation by tankyrase and prevents their inappropriate localization [13].

POT1 and TRF2 interact with each other and protect the telomeres from the DNA damage response triggered by the ATR and ATM pathways independently [14],[15]. POT1-deleted mice show increased p53-dependent apoptosis, as well as elevated DNA damage response and chromosomal fusions [16]. TPP1 forms a heterodimer with POT1 and acts as the bridge between the TRF1 complex and telomerase [17],[18].

## Telomeres and telomerase in hematologic malignancies

### Acute leukemia (AL)

AL is characterized by uncontrolled proliferation of myeloid precursor cells (acute myeloid leukemia, AML) or lymphoblast cells (acute lymphoblastic leukemia, ALL). Shortened telomere length is observed in patients with AL and is associated with chromosome instability. Capraro *et al*. reported that AL patients with an aberrant karyotype have significantly shorter telomere length than patients with a normal karyotype, and those patients with multiple aberrations possess the shortest telomeres. They also compared telomere length and telomerase activity in different subtypes of AL cells. They found that B-ALL cells had the shortest telomeres and the highest level of telomerase activity among all the subtypes of AL. Leukemia cells with abnormal karyotypes exhibited shorter telomeres than those with a normal karyotype (4.5 kb vs. 9.14 kb in ALL, 4.33 kb vs. 7.06 kb in AML) [19]. The order of telomerase activity is ranked B-ALL > AML > T-ALL.

The hTERT component of telomerase possesses many alternatively-spliced forms in which only the full-length transcript (+α + β) can be translated into a properly active enzyme. B-ALL cells exhibit the highest proportion of the full-length active product, in line with the trend in telomerase activity. Among all French-American-British (FAB) subtypes of AML, M0 and M3 have the lowest telomerase activity [19]. The telomerase activity of ALL cells has been reported to be higher in male patients with than in female patients. This may be due to the negative regulatory function of estrogen on telomerase [20].

Shortened telomere length and increased telomerase activity are associated with reduced response to chemotherapy, faster disease progression and poor prognosis in patients with AL. AML patients in the late stage of the disease have shorter telomere lengths and higher telomerase activity than those in the early stages. Relapsed AML patients have the shortest telomere length and highest telomerase activity [21],[22]. Asfour *et al*. reported that apoptosis of the leukemic blasts was blocked by telomerase in ALL patients, resulting in an increased tumor burden, faster disease progression and shortened survival [20]. Telomerase activities in ALL patients correlated to the enzyme concentration of lactate dehydrogenase (LDH), which is an adverse prognostic factor in ALL patients [20].

Altered expression of shelterin proteins has also been reported in *de novo* acute leukemic cells. Shi *et al*. reported that the expression of TRF1, which is a negative regulator of telomerase activity, was lower in patients with AL than in normal volunteers [23]. Ohyashiki *et al*. reported that patients with high hTERT expression or longer telomere length tended to have higher levels of TRF1 expression [24]. Capraro *et al*. compared the expression of shelterin proteins between different subtypes of AL. They found that TRF1 expression was reduced in AL and was lowest in AML patients. Meanwhile TRF2 expression was increased in AL and was highest in B-ALL patients, especially in B-ALL patients with abnormal karyotype. Expression of both TPP1 and RAP1 was increased in AL and was highest in T-ALL patients [19]. After chemotherapy, the TRF1 expression level increased in patients achieving complete remission (CR) but not in those patients in whom CR was not achieved [23]. When the acute promyelocytic leukemia (APL) cell line HL-60 was induced to differentiate into mature cells, telomerase activity was reduced and the expression of TRF1, TRF2 and TIN2 was increased [25].

### Chronic lymphocytic leukemia (CLL)

CLL is characterized by an abnormal expansion of mature B lymphocytes. Reliable prognostic factors are crucial for choosing therapeutic strategies in CLL patients and in predicting the outcome (reviewed by Kipps) [26]. Shorter telomeres, increased telomerase activity and altered expression of shelterin proteins are all observed in CLL patients. Patients with high-risk genomic aberrations, 11q or 17p deletions, undergo more severe telomere attrition due to the loss of ATM or p53, which are necessary to trigger p53-dependent apoptosis and the DNA damage response [27]. CLL patients with ATM mutations all display extreme telomere shortening independent of disease stage [28]. Patients with the chromosome aberration of a 17p deletion also show up-regulated c-myc which is a positive regulator of hTERT [29]. Functional assessment of the TP53 pathway was recently proposed as a method to precisely identify high risk CLL [30]. More evidence will be necessary to confirm the connection between telomere dysregulation and dysfunction of the TP53 pathway in CLL patients.

The observed shortening of telomere length in CLL patients was in line with other classical biological factors of CLL, including unmutated immunoglobulin variable region genes (UM-IGVH), CD38 and ZAP-70 positivity (>30%) and short (<6 months) lymphocyte doubling time [29]. Sellmann *et al*. reported a linear correlation between the frequency of IGHV gene mutations and the length of the telomeres [31]. CLL patients with shorter telomere length experienced worse clinical outcomes including shorter progression-free survival (PFS) and overall survival (OS) [29],[32]. Thus telomere length was indicated as a negative prognostic factor and a threshold of -4.2 kb was suggested to be a predictive separation in Sellmann’s study [31].

Shelterin alterations were also identified in CLL patients and were found to be involved in telomere instability. The expression levels of TRF1, RAP1 and POT1 were all reported to be reduced in B-CLL cells, while the expression of TPP1 was increased [33]. In contrast, the expression of TPP1 and TIN2 were reported to be down-regulated in newly diagnosed Binet stage A CLL patients in another study [34]. Telomere dysfunction caused by a POT1-encoding gene mutation was recently identified in CLL patients. The frequency of POT1 mutation was 3.5% in CLL patients and reached 9% in CLL patients without IGHV mutations [35].

### Chronic myelocytic leukemia (CML)

CML is a myeloproliferative neoplasm characterized by the presence of BCR/ABL fusion genes which encode the Bcr-Abl fusion protein. The tyrosine kinase activity of the Bcr-Abl fusion protein induces oxidative damage and telomere shortening by generating reactive oxygen species [36]. A shorter average telomere length was found in leukemic cells of CML patients compared with white blood cells of age-matched healthy individuals or BCR/ABL-negative T lymphocytes from the same patients [37]. Furthermore, telomere shortening was accelerated as the disease progressed from the chronic phase (CP) to the blastic phase (BP), with a rate approximately 10 times that in normal controls. Patients with a high-risk Hasford score at diagnosis exhibited significantly greater telomere loss than patients with a low-risk score, while patients with intermediate risk showed an intermediate telomere loss rate [38]. Average telomere length increased when patients achieved complete cytogenetic remission (CCR) under treatment with Imatinib Mesylate (IM) owing to the hematopoietic reconstruction of BCR/ABL negative cells. In contrast, patients who did not respond to IM exhibited a decrease in telomere length consistent with rapid cell division [38]. Braig *et al*. utilized bone marrow cells of telomerase knockout mice to establish a CML-like cell culture. They demonstrated the presence of shorted telomeres with an increase in the secretion of pro-inflammatory cytokines and growth factors associated with proliferation control in telomerase negative CML-like cells. Their work indicated that a telomerase-targeting strategy could induce senescence in CML-like cells and alleviate the tumor promoting/progressing effect of BCR-ABL [39]. Bakalova *et al*. demonstrated cross-talk between Bcr-Abl/c-Abl tyrosine kinase and telomerase in CML/ALL cells. Antisense inhibition of Bcr-Abl/c-Abl proteins was able to reactivate telomerase to maintain cell proliferation. Therefore the combination of TKI and telomerase targeting may be a promising strategy in CML treatment [40].

Individual telomere lengths have been determined in CML patients. Pronounced shortening of telomeres has been found on Yp, Yq, 1q, 5q, 9q, 8p, 21p and 21q. Genomic instability caused by high telomere attrition rates on the Y chromosome and on chromosome 21 might account for secondary chromosomal abnormalities during disease progression. An interesting finding was that telomere length in some individual chromosome ends was well maintained or even elongated. Some long-telomere chromosome arms (7q, 11p, 15p, and 18p) recurrently and specifically showed up in CML samples compared with healthy controls. Long telomeres on key chromosomes may contribute to a cell proliferation advantage during clonal selection in the early stage of CML ontogenesis [41],[42].

The circular extra-chromosomal telomeric repeat (ECTR), one of the ALT hallmarks, was used to define ALT activation in CML patients in a recent study. In this study, 27% of CML patients in CP were reported to exhibit both high ALT activity and telomerase activities. As telomerase activity increases with disease progression, the dominating telomere maintenance mechanism might transition from ALT to telomerase [43].

Altered expression of shelterin proteins was also reported in progressing CML patients. Campbell *et al*. reported that expression levels of TRF1 and TRF2 were increased in CML patients in the CP and in the accelerated phase (AP) but reduced to a level comparable to normal controls in the majority of patients in the BP. Increased expression of TRF1 may thus induce telomere shortening in CML in both the CP and AP [44]. High levels of TRF2 and tankyrase might contribute to the delay in senescence signaling triggered by the critically shortened telomeres and the consequent maintenance of telomere length. When K562 cells were treated with Anti-Bcr-Abl mRNA, decreased expression of TRF1 and TIN2 were observed [40].

### Myelodysplastic syndromes (MDS)

MDS are a group of disorders characterized by dysplastic features in the hematopoietic cells with the tendency to progress into acute leukemia [45]. By reason of the heterogeneity of the diseases, results of telomere length of MDS patients are not consistent. Recently, shortened telomeres have been reported in isolated peripheral blood leukocytes, CD15+ myeloid cells and CD19+ lymphocytes of MDS patients [46]. Although the MDS clone originates within the myeloid compartment, abolishment of T-lymphocytes differentiation and loss of naïve T-cells are consequences of hTERT deficiency [47]. However, telomere lengths in marrow stromal cells are reported to be stabilized well [48]. The telomere lengths of individual chromosome arms was measured by Lange *et al*., who found markedly longer telomeres in several chromosome arms in patients with an isolated monosomy 7 compared with patients with a normal karyotype or a complex karyotype [49]. The mechanism(s) involved in the stabilization or elongation of telomeres in patients with monosomy 7 still requires future exploration.

Patients with telomere shortening at the time of diagnosis showed a high frequency of complex chromosome abnormalities, faster disease evolution and shorter survival time [50],[51]. Sieglová *et al*. compared the telomere length of MDS patients with different FAB subtypes. Telomere length in the early phases of MDS (RA and RARS) was longer compared to that in advanced forms of MDS (RAEB + RAEB-T) and was shortest in secondary AML from MDS. A significant negative correlation between telomere length in bone marrow cells of MDS patients and the International Prognostic Score System (IPSS) score was observed [52]. In order to stabilize telomere length, hTERT expression level and telomerase activity were increased in MDS patients in the more advanced stages [53]. Thus shortened telomeres and increased telomerase activity could be regarded as prognostic factors for MDS patients. In a recent study, three-dimensional quantitative FISH of telomeres was carried out on nuclei from bone marrow samples of MDS and AML patients. Three-dimensional (3D) telomeric profiles which were determined based on the nuclear telomeric architecture, telomere numbers, the presence of telomere aggregates, telomere signal intensities, nuclear volumes, and nuclear telomere distribution, confirmed a dynamic and evolutionary process of telomere dysfunction during the transformation of MDS to AML [54].

## Telomeres and telomerase in hematopoietic stem cell transplantation (HSCT)

HSCT is a potential curative therapy for many hematologic disorders and immunodeficiency diseases. The self-renewal capacity of hematopoietic stem cells (HSCs) is essential for reconstitution of the hematopoietic system. The proliferative potential of HSCs decreases with differentiation and age in line with the shortening of telomeres and increased telomerase activities in these cells. Mean telomere length is shorter in HSCs purified from adult bone marrow than from fetal liver or umbilical cord blood [55]. Telomerase activity is higher in HSCs during the late development stage than in non-expanding HSCs [56].

hTERT-deleted mice have been used as a telomerase-deficient model for the study of telomerase function in the hematopoietic system [57]–[59]. Sekulovic *et al*. reported the loss of a 10 kb length of the telomere of leukocytes generated from hTERT knockout HSCs after 6 days of *in vitro* expansion and 3 months of regeneration in secondary-transplanted recipient mice [57]. Telomere dysfunction impaired mesenchymal progenitor cell function, reducing the capacity of bone marrow stromal cells for maintaining functional HSCs. When wild-type HSCs were transplanted into TERC-knockout recipient mice, accelerated myelopoiesis and impaired B-cell development occurred [59].

Patients who received autologous or allogeneic HSCT (auto-HSCT or allo-HSCT) experienced more severe erosion of telomere length in their blood cells under massive differentiation pressure compared with their donors. Akiyama *et al*. reported that the telomeres of transplanted cells became shortened by up to 1.9 kb in auto-HSCT recipients over an observation period of 5.3 years, the same frequency of telomere erosion as would occur over 15–20 years in normal individuals. Telomere erosions of up to 2.1 kb were observed in patients who received allo-HSCT [60]. Baerlocher *et al*. evaluated 44 long-term survivors after allo-HSCT with a median follow-up of 17.5 years. Significantly shortened telomere length was observed in all blood cells lineages, including granulocytes, naïve/memory T cells, B cells and natural killer/natural killer T cells in the recipients compared with that in their donors [61]. The rate of telomere shortening in recipients is highest in the first year after HSCT and then slows down to a rate similar to that of their donor and of healthy controls [62]. Telomere shortening in patients who received allo-HSCT seems to be more sensitive to the influence of ageing than auto-HSCT. A correlation between donor age and telomere shortening rate has been found in allo-HSCT but not in auto-HSCT recipients [60].

In allo-HSCT, more severe telomere shortening is associated with elderly donors, female donors and the development of chronic graft-versus-host disease (cGVHD) [60],[61]. The accelerated telomere shortening of transplanted female donor cells may be a consequence of the deficiency of estrogen-upregulated telomerase activity after transplantation [63]. Patients who develop cGVHD also show more severe telomere attrition, probably due to chronic inflammation and oxidative stress. In contrast, development of acute GVHD (aGVHD) has no significant impact on telomere length in recipients [61]. CD4 + CD25 + Foxp3+ regulatory T cells (Treg) constitute a lymphocyte subgroup responsible for the control of cGVHD. Patients with telomerase deficiency in Treg have impaired proliferative capacity of these cells and consequently a higher incidence of moderate or severe cGVHD [64].

**Figure 2:**
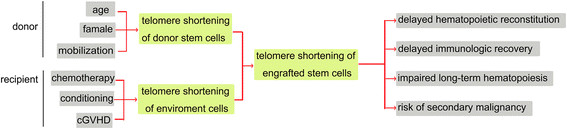
**Factors affecting telomere length and recipient outcomes in hematopoietic stem cell transplantation.** Mobilization, older donors, and female donors are related to shortened telomeres in donor stem cells. Chemotherapy and conditioning courses before hematopoietic stem cell transplantation (HSCT) could also accelerate telomere shortening in host cells. The telomere length (TL) of grafted stem cells is mainly determined by the pretransplantation TL of donor cells, but is also affected by the host environment and by the occurrence of chronic graft versus host disease (cGVHD). The TL of engrafted donor stem cells is important for the continuous and stable reconstitution of the hematopoietic system.

Treatments involved in the HSCT procedure could also have an impact on the telomere homeostasis of engrafted HSCs and may eventually influence the outcomes of recipients (Figure 2). In patients who received auto-HSCT, pre-transplantation chemotherapy has a significant influence on the telomere length of transplanted cells. Telomere length is negatively related to the number of courses of cytoreductive therapy received by patients [60]. Ricca *et al*. compared telomere length in peripheral blood progenitor cells (PBSCs) collected after two tightly-spaced high-dose (hd) chemotherapy courses. Telomere length was significantly shorter in PBSCs collected after the second course (hd-Ara-C) compared to that collected after the first course (hd-CY) [65]. This difference in telomere length of collected PBSCs determines the telomere length of the hematopoietic cells after auto-HSCT. In another study patients transplanted with PBSCs from the second collection had significantly shorter telomeres than those who received PBSCs from the first collection [66].

Donors’ HSCs with longer telomeres could offer a replicative advantage and lead to faster granulocyte recovery in the recipient after HSCT. In contrast, patients with shorter telomere lengths after HSCT took a longer period to reach neutrophil recovery and had a greater risk of developing hematopoietic disorders [67]. Accelerated telomere shortening and consequent chromosomal instability are independently associated with the development of therapy-related myelodysplasia or acute myelogenous leukemia (t-MDS/AML) after auto-HSCT in patients with Hodgkin’s lymphoma or non-Hodgkin’s lymphoma. In patients who developed t-MDS/AML, reduced proliferative capacity of HSCs contributed to decreased generation of committed progenitors [68].

The telomere length of hematopoietic cells in the recipient before HSCT, which represents the inner environment of host, is another factor impacting the outcome of transplantation. Peffault *et al*. reported that treatment-related mortality was inversely correlated with age-adjusted recipients’ pre-transplantation telomere length in their lymphocytes (hazard ratio, 0.4) in patients who received allo-HSCT, especially in patients with advanced stage disease [69].

## Telomerase-targeting therapies

**Figure 3:**
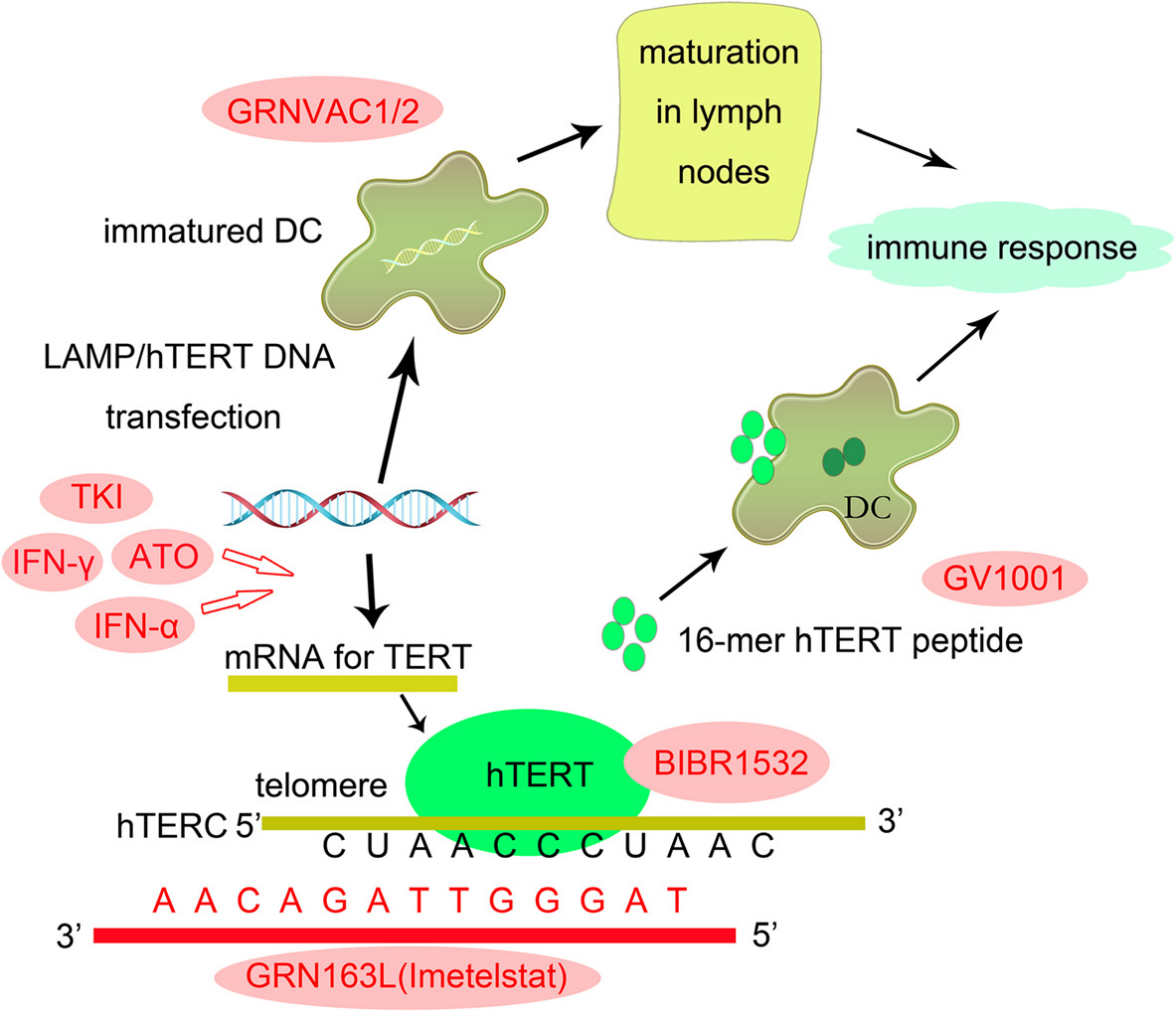
**Telomerase inhibition therapies utilized in the treatment of hematologic malignancies.** Telomerase-based immunotherapies utilize telomerase-associated antigens to produce an immune response which ultimately leads to lysis of tumor cells. Tyrosine kinase inhibitors (TKI), arsenic trioxide (ATO), interferon alpha (IFN-α) and interferon gamma (IFN-γ) reduce the expression of hTERT; imetelstat (GRN163) is complementary to the template region of the telomerase RNA component hTERC, competitively binding and blocking telomerase; BIBR1532 inhibits the specific active site of hTERT.

Since the increased expression of hTERT and activation of telomerase are universally involved in oncogenesis and the progression of hematopoietic malignancies, telomerase inhibition could be an effective antineoplastic strategy for therapy (Figure 3).

### Immunotherapies

Telomerase-based immunotherapies utilize telomerase-associated antigens to elicit CD4+ and CD8+ T-cell responses and the cytotoxic T lymphocyte (CTL) response, which ultimately lead to lysis of tumor cells.

GV1001 is an MHC class II-restricted hTERT peptide vaccine, which consists of amino acids 611–626 of the hTERT active site. Granulocyte-monocyte colony-stimulating factor (GM-CSF) and Toll-like receptor-7 (TLR-7) have been used as adjuvants in the GV1001 vaccine to eliminate the problem of self-tolerance. Preclinical studies in B-CLL patients confirmed that telomerase-positive leukemic cells can naturally induce telomerase-specific T cells [70]. However, a recent randomized phase III clinical trials reported that combining GV1001 to gemcitabine and capecitabine did not improve the overall survival of patients with locally advanced or metastatic pancreatic cancer [71].

Another vaccine, Vx-001, consists of a cryptic peptide of hTERT or its optimized version with a modified tyrosine (Y1) residue at the first amino-acid and enhanced peptide affinity. The efficacy and safety of Vx-001 has been confirmed in mouse model and in phase I/II clinical trials in patients with various types of tumors. This vaccine is now scheduled for testing in a phase III clinical trial in NSCLC patients [72]–[74].

GRNVAC1 is a dendritic cell (DC) -based telomerase-associated vaccine. Immature DCs are mobilized and isolated from the patient’s own peripheral blood then transduced with mRNAs encoding the full-length hTERT protein *in vitro*. The lysosome-associated membrane protein (LAMP-1) mRNA sequence is co-transduced into DCs to make the antigen easily degradable and to enhance the immune response [75]. GRNVAC1 was tested in a randomized phase II clinical trial in patients with AML. Prolonged vaccination (up to 32 administrations) of GRNVAC1 in AML patients was well tolerated in most patients, and produced a greater effect in AML patients at high risk of relapse [76],[77].

### Antisense oligonucleotide

GRN163L (Imetelstat) is the most promising oligonucleotide possessing the ability to block telomerase by acting as a complementary sequence to hTERC. In an *in vitro* study, GRN163L showed effective inhibition of telomerase and of cell growth in B-CLL cells and tumor initiating B cells of patients with multiple myeloma (MM) [78],[79]. GRN163L has been entered into stage I and II clinical trials in patients with refractory and relapsed MM and some types of solid tumors. GRN163L was reported to be generally well-tolerated in patients with relapsed and refractory MM. The most common treatment-related event was thrombocytopenia and prolongation of the activated partial thromboplastin time (APTT). The most marked hematologic toxicity was observed in two patients with prior auto-HSCT [80]. That may have been due to blockage of telomerase activity in HSCs which impaired reconstitution of the hematologic system. In a recent single-center study, GRN163L was shown to be effective in inducing morphologic and molecular remissions in patients with myelofibrosis, with a response rate of 44% [81].

### BIBR1532

BIBR1532 is a synthetic non-nucleotidic small molecule which selectively inhibits the active site of telomerase. BIBR1532 leads to progressive telomere shortening and apoptotic cell death in a concentration-dependent manner in AML cell lines as well as in primary cells from patients with AML or CLL [82]–[84]. BIBR1532 inhibits the activity of telomerase through transcriptional suppression of survivin-mediated c-Myc and hTERT expression, increasing p73 and p21 expression, up-regulating the Bax/Bcl-2 molecular ratio and finally increasing P53-induced apoptosis [84],[85]. P53 is the final executant of the telomerase-inhibiting effect of BIBR1532. In P53-negative K562 cells, the telomere length was stabilized when it reached approximately 5 kb [85].

### Other drugs with telomerase inhibiting activity

IM (Gleevec), the first selective tyrosine kinase inhibitors (TKI), is reported to cause a dose-dependent inhibition of telomerase activity in various leukemia cell lines, including BCR-ABL negative cell lines [86]–[88]. IM regulates telomerase activity by decreasing the expression of hTERT and increasing the expression of telomerase inhibitor protein phosphatase 2A (PP2A) [87]. Following treatment with IM, the expression levels of TRF1, TRF2 and PinX1 are markedly reduced. The second-generation TKIs nilotinib and dasatinib, which have higher potency than imatinib against BCR-ABL (reviewed by Wei *et al*.), are more effective in reducing telomerase activity [89],[90].

Arsenic trioxide (ATO) is successfully used to induce complete remission and to trigger apoptotic death of APL cells [91],[92]. Ghaffari *et al*. reported a dose-dependent inhibition of telomerase activity of ATO and a reduction in telomere length in ATO-treated NB4 cells. The mRNA levels of Pin1, survivin, c-Myc, hTERT, and PinX1 were all reduced in a concentration-dependent manner after 2 days of ATO treatment [93].

Interferons (IFNs) are multi-functional cytokines produced by eukaryotic cells. Xu *et al*. reported that IFN-α could significantly down-regulate the expression of hTERT and the activity of telomerase in many types of human hematologic malignant cell lines, primary leukemic cells and T-lymphocytes within 4 hours of treatment at a concentration of 5000 U/mL, through suppressing the hTERT promoter activity [94]. Lindkvist *et al*. reported that IFN-γ could also induce a decrease of hTERT expression. hTERT mRNA levels were virtually abolished after 48 h of IFN-γ treatment at 5000 U/mL [95].

## Conclusions

Telomeres are essential for the maintenance of chromosome stability in mammalian cells. Accelerated telomere shortening leads to activation of telomerase in stem cells and in the majority of tumor cells. In patients with hematologic malignancies, shortened telomeres and increased telomerase activity are usually observed and are associated with disease progression. In patients who have received HSCT the telomere length of engrafted stem cells is closely related to the outcomes of HSCT. Consequently the telomere characteristics should be taken into consideration during donor selection. It is also necessary to evaluate the effect of chemotherapy and conditioning courses on telomere length. Many promising telomerase targeting therapies have been confirmed to be tolerable and efficient to induce immune responeses in patients with hematological malignancies. However optimized strategies are still required to ensure their clinical efficiency. Further work will be needed to elucidate the complete story of telomere biology and to explore efficient telomerase-targeting therapies in hematologic malignances.

## Abbreviations

ATM: Ataxia telangiectasia-mutated gene

ATR: Ataxia telangiectasia and Rad3

ALT: Alternative lengthening of telomeres

ss DNA: Single-strand DNA

ds DNA: Double-strand DNA

TRF1: Telomeric repeat-binding factors 1

TRF2: Telomeric repeat-binding factors 2

TIN2: TRF1-interacting nuclear factor 2

Rap1: TRF2-interacting protein 1

POT1: Protection of telomeres protein 1

TPP1: POT1 and TIN2-interacting protein 1

AL: Acute leukemia

AML: Acute myeloid leukemia

ALL: Acute lymphoblastic leukemia

LDH: Lactate dehydrogenase

CR: Complete remission

APL: Acute promyelocytic leukemia

CLL: Chronic lymphocytic leukemia

UM-IGVH: Unmutated immunoglobulin variable region

PFS: Progression-free survival

OS: Overall survival

CML: Chronic myelocytic leukemia

CP: Chronic phase

BP: Blastic phase

CCR: Completely cytogenetic remission

IM: Imatinib Mesylate

ECTR: Extra-chromosomal telomeric repeat

AP: Accelerated phase

MDS: Myelodysplastic syndromes

IPSS: International Prognostic Score System

3D: Three-dimensional

HSCT: Hematopoietic stem cell transplantation

HSCs: Hematopoietic stem cells

auto-HSCT: Autologous hematopoietic stem cell transplantation

allo-HSCT: Allogeneic hematopoietic stem cell transplantation

GVHD: Graft-versus-host disease

cGVHD: Chronic graft-versus-host disease

aGVHD: Acute graft-versus-host disease

Treg: Regulatory T cells

PBSCs: Peripheral blood progenitor cells

t-MDS/AML: Therapy-related myelodysplasia or acute myelogenous leukemia

CTL: Cytotoxic T lymphocyte

GM-CSF: Granulocyte-monocyte colony-stimulating factor

TLR-7: Toll-like receptor-7

DC: Dendritic cell

MM: Multiple myeloma

APTT: Activated partial thromboplastin time

TKI: Tyrosine kinase inhibitors

PP2A: Protein phosphatase 2A

ATO: Arsenic trioxide

## Competing interests

The authors declare no conflict of interest.

## Authors’ contributions

LW participated in the design of this review, performed the selection and interpretation of data, and drafted the manuscript. HX participated in the design of this review, helped to draft and revise the manuscript. XZ performed the selection and interpretation of information about acute leukemia and helped to draft the manuscript. CW performed the selection and interpretation of information about the telomere biology and helped to draft the manuscript. HH conceived of the review, and participated in its design and coordination and revised the manuscript. All authors read and approved the final manuscript.
